# Effects of pasture consumption and obesity on insulin dysregulation and adiponectin concentrations in UK native‐breed ponies

**DOI:** 10.1111/evj.14507

**Published:** 2025-04-21

**Authors:** Marine A. Barnabé, Jonathan Elliott, Patricia A. Harris, Nicola J. Menzies‐Gow

**Affiliations:** ^1^ Department of Clinical Sciences and Services Royal Veterinary College Hertfordshire UK; ^2^ Department of Comparative Biomedical Sciences Royal Veterinary College Hertfordshire UK; ^3^ Equine Studies Group Waltham Petcare Science Institute Leicestershire UK

**Keywords:** adiponectin, body condition score, equine metabolic syndrome, horse, insulin dysregulation, laminitis, pony

## Abstract

**Background:**

Insulin dysregulation (ID) and hypoadiponectinaemia (total [adiponectin] <7.9 μg/mL) are risk factors for laminitis. They are sometimes, but not always, associated with obesity.

**Objectives:**

To investigate the effects of pasture consumption and obesity on ID and circulating total [adiponectin] in ponies.

**Study Design:**

Longitudinal.

**Methods:**

Seven native‐breed ponies with normal basal and post‐oral sugar test (OST) [insulin] and body condition score (BCS) 4.3–5.5/9 were allowed to graze until they reached BCS 7/9. Ponies were then maintained at BCS 7/9 until completion of the study (week 22). Morphometric measures, OST, insulin tolerance test (ITT), plasma [adiponectin], whole‐blood expression of receptors for adiponectin, insulin, and insulin‐like growth factor 1, and pasture conditions (height and vigour) were determined fortnightly.

**Results:**

Median (range) BCS increased significantly (*p* < 0.001) from 5.0 (4.3–5.5; week 0) to 7.2 (5.7–7.5; week 22). Basal [insulin] did not change significantly over the study, but median post‐OST [insulin] was significantly higher (*p* < 0.05) at week 14 (95.2 [17.9–114.0] μIU/mL), week 16 (103.0 [16.4–166.0] μIU/mL), and week 20 (93.6 [10.0–153.0] μIU/mL) than week 0 (25.0 [10.0–64.0] μIU/mL). Compared with week 0, ITT results were significantly lower at weeks 2–6 and 12–20, and [adiponectin] was significantly lower at weeks 10–22 (*p* < 0.05). [Adiponectin] decreased in all ponies during the study. Both low (3/10) and high (8–9/10) pasture scores were significantly associated with low ITT results. Low pasture scores were associated with low [adiponectin]. BCS was significantly associated with basal [insulin], post‐OST [insulin], ITT results, but not [adiponectin].

**Main Limitations:**

No control group with maintenance of ideal BCS; small sample size comprising native UK ponies.

**Conclusions:**

Six ponies developed hypoadiponectinaemia, and all showed transient or consistent ID during the study. Both short, stressed grass and long, lush grass were associated with decreased tissue insulin sensitivity.

## Resumen

**Historial:**

La desregulación insulínica (ID) y la hipoadiponectinemia ([adiponectina]total < 7.9 μg/mL) son factores de riesgo para laminitis. Se asocian, algunas veces, pero no siempre, con obesidad.

**Objetivos:**

Investigar los efectos del consumo de pasto y obesidad sobre la ID y la [adiponectina] total en ponis.

**Métodos:**

Siete ponis de razas nativas con una prueba oral basal y post glucosa normales (OST) de [insulina] y clasificación de condición corporal (BCS) 4.3–5.5/9, fueron permitidos pastar hasta que llegasen a tener un BCS 7/9. Los ponis luego fueron mantenidos en BCS 7/9 hasta completar el estudio (semana 22). Medidas morfométricas, OST, prueba a la tolerancia a la insulina (ITT), [adiponectina] plasmática, expresión de receptores en sangre entera a adiponectina, insulina, and factor 1 de crecimiento tipo insulina, y condiciones del pasto (altura y vigor) fueron medidos cada 2 semanas.

**Resultados:**

La mediana de los BCS (rango) aumento significativamente (P<0.001) de 5.0 (4.3–5.5; semana 0) a 7.2 (5.7–7.5; semana 22). [Insulina] basal no cambio significativamente durante el estudio pero la mediana post‐OST [insulina] fue significativamente mayor (P<0.05) en la semana 14 (95.2 [17.9–114.0] μIU/mL), semana 16 (103.0 [16.4–166.0] μIU/mL), y semana 20 (93.6 [10.0–153.0] μIU/mL) que la semana 0 (25.0 [10.0–64.0] μIU/mL). En comparación con la semana 0, los resultados de ITT fueron significativamente menores en las semana 2–6 y 12–20, y [adiponectina] fue significativamente menor a las semanas 10–22 (P<0.05). La [adiponectina] disminuyó en todos los ponis durante el estudio. Tanto los niveles bajos (3/10) y altos (8‐9/10) de pasto, fueron significativamente asociados con resultados de ITT bajos. Niveles bajos de pasto fueron asociados con bajas [adiponectina]. Los BCS fueron asociados significativamente con [insulina] basal, [insulina] post‐OST, resultados de ITT, pero no con [adiponectina].

**Limitaciones principales:**

No hay grupo control con mantención de BCS ideales; tamaño de muestra pequeño que incluye razas nativas del RU.

**Conclusiones:**

Seis ponis desarrollaron hipoadiponectemia y todos mostraron una ID transitoria o consistente durante el estudio. Tanto el pasto corto, estresado y aquel largo y frondoso, se asocio con una reducción en la capacidad tisular de respuesta a la insulina.

## 1 INTRODUCTION

Insulin dysregulation (ID), which may present as tissue insulin resistance (IR), basal, or post‐prandial hyperinsulinaemia, is the central defining feature of the equine metabolic syndrome (EMS).^1^ The most important consequence of EMS is the development of endocrinopathic laminitis, a painful and potentially devastating condition that can lead to euthanasia if it becomes chronic or cannot be adequately managed in affected equids. Obesity can contribute to ID^2^ and is a commonly associated finding in equids with EMS.^1^ As well as its role in ID and EMS, equine obesity is a serious and highly prevalent health issue affecting up to 60% of the UK leisure horse population.^3^ In addition to ID, hypoadiponectinaemia is another important independent risk factor for equine endocrinopathic laminitis and is sometimes a component of EMS.^4^, ^5^ The complex relationships between ID, hypoadiponectinaemia, and obesity remain unclear, with studies reporting varying and sometimes conflicting observations.

In previous studies, subjective measures of obesity such as body condition score (BCS)^6^ and cresty neck score (CNS)^7^ were negatively associated with high molecular‐weight adiponectin concentrations. In addition, CNS was positively associated with both basal and post‐prandial insulin concentrations,^7^ and BCS with basal insulin.^6^ However, the positive relationship between obesity, ID, and hypoadiponectinaemia is not a consistent finding in all studies.^8^, ^9^ ID and hypoadiponectinaemia are not always consequences of obesity and can also occur in lean animals, just as obese animals can have normal total adiponectin concentrations and insulin sensitivity status.^8^, ^9^, ^10^ Furthermore, previous research has shown that changes in plasma adiponectin concentrations were more closely linked to dietary composition than intake.^10^ Indeed, ponies that became obese after consuming a high‐starch diet showed lower insulin sensitivity and lower plasma adiponectin concentrations than those who had become obese by consuming a high‐fat diet^10^ or a once‐daily high‐glycaemic meal.^8^ Finally, previous work has shown that short‐term, experimentally induced ID did not lead to changes in circulating total adiponectin concentrations, raising further questions about the relationship between ID and hypoadiponectinaemia.^11^

The present study aimed to investigate the effects of pasture‐induced weight gain and maintained obesity on ID and circulating total adiponectin concentrations ([adiponectin]) in non‐laminitic, UK native‐breed ponies. Animals had normal basal and post‐oral sugar test (OST) [insulin] and were of ideal weight at the start of the study. Weight gain was induced by providing access to late spring/summer grazing as an exclusive diet, as this reflects the living conditions of many ponies in the UK kept at pasture over the spring/summer months.^12^ Although similar studies have investigated the effects of weight gain induced by high‐fat and high‐glycaemic diets,^8^, ^10^ this is the first study focused on weight gain induced solely by pasture consumption. We hypothesised that weight gain would be associated with decreased [adiponectin] and the development of ID in some or all animals.

In addition to this primary aim, changes in the expression of receptors for adiponectin (AdipoR1/2), insulin (INSR), and insulin‐like growth factor 1 (IGF‐1R) were investigated and it was hypothesised that any changes in [insulin] and [adiponectin] would be associated with physiologically appropriate regulation of the respective receptor expression. Adiponectin undergoes post‐translational modifications to produce different isoforms with different affinities to each receptor, leading to different downstream signalling pathways. Although there are currently no validated assays available to measure these adiponectin isoforms in the UK, measuring the expression of its two receptors may provide some insight into the regulation of different adiponectin signalling pathways. Receptors for insulin and IGF‐1 were included because of the hypothesis linking inappropriate activation of IGF‐1R by insulin to the pathogenesis of endocrinopathic laminitis.^13^

Finally, a simple pasture scoring system was designed for potential use by horse‐owners. Associations between pasture scores, morphometric, and metabolic measures were investigated to determine if visual inspection of pasture conditions could provide some insight into associated changes in weight, [adiponectin], and ID status.

## 2 METHODS

### 2.1 Study overview

This study was conducted between 16 May 2022 and 19 October 2022 in Hertfordshire, south‐east England. Healthy ponies of ideal weight were turned out to pasture of a sufficient herbage to naturally induce weight gain. Morphometry, metabolic parameters, and pasture scores were recorded every 2 weeks, with the first week of the study referred to as week 0 and assessments carried out on all even‐numbered weeks, ending on week 22. Each time BCS and weight were assessed, paddock fencing was moved to provide more or less grazing as appropriate to ensure all ponies gained weight gradually. It was anticipated that ponies would take 9–12 weeks to reach BCS 7/9, according to previous unpublished work. Once ponies reached BCS 7/9, access to the grazing area was continually adjusted so that animals would maintain this BCS, but not increase any further to minimise the risk of developing laminitis. Animals were checked daily to monitor any early signs of laminitis such as lameness, reluctance to move/turn, digital pulses, and changes in demeanour.

### 2.2 Sample size calculation

Previous work showed that feeding a diet rich in starch to induce obesity in ponies resulted in a 45% decrease in insulin sensitivity.^10^ Median [adiponectin] measured using a validated immunoturbidimetric assay in normal ponies is 3.72 (2.55–5.06) μg/mL,^5^ and a sample size calculation assuming 80% power and *p* = 0.05 therefore suggested that *n* = 6 ponies would be sufficient to detect a similar magnitude reduction in insulin sensitivity and/or a 25% decrease in plasma [adiponectin] when inducing obesity. To mitigate for any potential reduction in numbers (e.g., ponies failing to gain weight or being removed from the study for any other reason), a total of 8 ponies were recruited to the study.

### 2.3 Animals

In total, eight non‐laminitic, native‐breed ponies (four mares and four geldings, aged 5–18 years) with ideal BCS (4.3–5.5/9)^14^ were initially included in this study. Animals had normal basal [insulin] (mean ± SD = 3.8 ± 2.2 μIU/mL; measured on Immulite 2000 xpi), and normal insulin responses (mean ± SD = 34.8 ± 14.8 μIU/mL) to a 60‐min OST performed with 0.45 mL/kg Karo Light Corn syrup.^15^ Adrenocorticotropic hormone concentrations were measured in animals aged >10 years and were within the season‐adjusted reference range.^16^ Animals were examined by an equine veterinarian and showed no clinical signs of acute or chronic laminitis (such as lameness, reluctance to more or turn, digital pulses, divergent hoof rings) or pituitary pars intermedia dysfunction (such as hypertrichosis or muscle loss^16^). Further details of the animals' signalment are provided in Table S1. Ponies were kept at an animal welfare charity for several months before the start of this study. They were kept on maintenance pasture and experienced similar management during this time. At the end of the study, ponies were returned to the charity and rehomed to members of the public through the organisation's usual processes.

Seven ponies (A–G) initially started the study at week 0 and five of these (B, C, D, F, G) completed the study in full, up to week 22. Pony E showed clinical signs of mild laminitis in week 5 and was removed from the study. Data from this pony were not included in any statistical analysis. In addition, pony A was removed from the study due to the development of mild laminitis during week 19. All available data from pony A were included. Finally, pony H joined the study at week 4 and went on to complete the study at week 22. For this pony, data from weeks 0 and 2 are treated as ‘missing’ and data from all other weeks are analysed with those from the other ponies in the study.

### 2.4 Morphometry

Body weight was determined using a calibrated weighbridge (Equestrian Weight Platform, Equestrian Products).^4^ CNS (0–5)^7^ and BCS were estimated (Kohnke‐modified Henneke scale 1–9)^14^, ^17^ by a single trained assessor. For BCS, scores were allocated to each of six body areas (neck, withers, loin, tailhead, ribs, and shoulders) using visual assessment and palpation and were averaged to determine the overall BCS. Morphometric measurements including heart girth, belly girth, and rump width were determined using a tape measure. Cumulative (relative to week 0), fortnightly changes (relative to measurements at the previous time‐point), and coefficient of variation (%CV) in these measures were calculated.

### 2.5 Metabolic parameters

OSTs and insulin tolerance tests (ITTs) were performed every 2 weeks on consecutive days to assess insulin response to oral hydrolysable carbohydrates and peripheral tissue IR, respectively. The OST was performed the day after weighing, and the ITT was performed the following day. This order was maintained throughout the study for consistency.

For the OST, baseline blood samples (T0) were collected via jugular venipuncture from non‐fasted animals^18^ into both plain and EDTA‐coated tubes, before oral administration of 0.45 mL/kg Karo Light Syrup using a catheter‐tip syringe.^15^ A second blood sample was collected after 60 min (T60). Animals remained at pasture throughout. Samples in plain tubes were left to clot at room temperature for at least 30 min before centrifuging at 3000*g* for 5 min to obtain serum. Blood samples collected into EDTA‐coated tubes were inverted several times and placed on ice before centrifuging at 500*g* for 5 min to obtain plasma. Serum [insulin] and plasma [adiponectin] were determined using assays validated for equine samples at a commercial laboratory (Liphook Equine Hospital Laboratory).^4^, ^15^, ^19^ T0 samples were used to determine basal [insulin] and [adiponectin], and T60 serum samples were used to determine insulin responses to oral sugars. Samples from week 0 to 6 were submitted for analysis in a single batch. Samples collected during subsequent weeks were submitted for analysis within 3 days of collection to ensure that data of the ponies' [insulin] were available as soon as possible following sampling. In addition, T60 plasma samples from weeks 0 to 8 were analysed to determine the short‐term effect of oral sugars on [adiponectin].

For the ITT, baseline blood samples (T0) were collected from non‐fasted animals via jugular venipuncture into plain tubes before administering 0.1 IU/kg insulin (Actrapid, Novo Nordisk) intravenously. A second blood sample was collected after 30 min (T30). Basal blood glucose concentrations were determined before insulin administration and at T30 using a handheld glucometer (Accu‐Chek Aviva, Roche) validated for equine samples.^20^ Animals remained at pasture throughout and were administered 100–200 mL Karo Light Syrup orally immediately after collection of the T30 blood sample if their blood glucose concentration was ≤2.0 mmol/L.

### 2.6 Definition of insulin dysregulation

Three manifestations of ID were defined according to the following criteria^15^:

- Basal hyperinsulinaemia: T0 [insulin] >31 μIU/mL.
- Excessive insulin response to oral sugars: T60 [insulin] >63 μIU/mL (OST results).
- Tissue IR: percentage decrease in blood glucose <50% (ITT results).

It is important to note that the threshold values for post‐OST [insulin] measured using the Immulite 2000 xpi recommended by the Equine Endocrinology Group (EEG)^15^ were determined in animals fasted before performing an OST. Animals were not fasted in the present study and were kept on pasture. A previous study has shown that allowing ponies to remain at pasture does not significantly alter OST results when compared with fasted animals.^21^ We also note that the EEG guidelines describe insulin concentrations of 30–75 μIU/mL as ‘suspect’ if consistent with clinical signs of ID, but we have chosen to use the lower, more conservative value of 31 μIU/mL to define basal hyperinsulinaemia. This is equivalent to the value used in a recent study by Clark et al. comparing the diagnostic value of different tests for ID.^22^ We acknowledge that other threshold values for basal and post‐OST hyperinsulinaemia have been proposed and may be more appropriate for predicting laminitis risk (rather than diagnosing ID) or for use in clinical practice.^4^

Hypoadiponectinaemia was defined as total [adiponectin] <7.9 μg/mL.^5^, ^15^

The percentage time each pony was insulin‐dysregulated (ID+) during the study was calculated according to the following formula:

$$\text{Percentage time pony was}\;{\mathrm{ID}}^{+}=\frac{\text{number of time points with}\;\mathrm{ID}}{\text{total number of time points with available data}}\times 100,$$

where ID is defined as meeting the criteria for any one of the three manifestations defined above.

### 2.7 Receptor expression

T0 blood samples collected during the OST were used to assess the RNA expression of AdipoR1, AdipoR2, INSR, and IGF‐1R in whole‐blood. Samples were processed and data were analysed according to methods described in Barnabé et al.^11^

### 2.8 Pasture scoring and weather data

The pasture was photographed 2 weeks before the start of the study and every 2 weeks on the day of the OST. Pasture conditions were assigned two scores (1–5): one for plant height (as an indicator of the total availability of forage) and one for vigour (as an indicator of plant health and growth phase; Table 1).^23^ These two scores were then added to obtain a combined score out of 10. In addition, weather data (monthly/weekly rainfall, sunshine hours, mean temperatures, and mean maximal temperatures) for England South‐East and Central South were retrieved from the Met Office^24^ and the governmental Environment Agency.^25^

**TABLE 1:** Pasture scoring system.^a^

Score	1	2	3	4	5
Height	Very short (<1 cm)	Short (1–5 cm)	Medium (6–10 cm)	Long (11–15 cm)	Very long (>15 cm)
Vigour	Very dry, yellow or brown in colour. Grass has the appearance of hay. Growth is severely stunted. 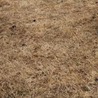	Yellowish foliage, some stunting evident. 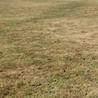	Green foliage, may have areas of yellow foliage due to manure/urine patches or dry swards. 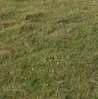	Light and dark green foliage. Evidence of growth. 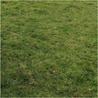	Very healthy, lush, dark green foliage. Actively growing. 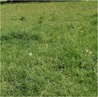

^a^ Scoring system adapted from the US Department of Agriculture's *Guide to Pasture Condition Scoring*.^23^ Photographs were taken by AUTHOR.

### 2.9 Analysis of factors associated with the development of insulin dysregulation

The distribution of morphometric, blood analyte, and weather data was assessed visually using histograms, and Shapiro–Wilk tests were performed.^26^ Correlations between different morphometric measurements were assessed using Spearman's correlation coefficient (GraphPad Prism v.9.1.2). Associations between pasture scores and weather data were investigated using Spearman's correlation coefficient (Prism v.9.1.2) and linear regression (SPSS v.29.0, IBM). Changes in morphometric measures and the concentrations of blood analytes over time were analysed using repeated measures models, with the pony as the subject variable and time (week) as the repeated measure (SPSS v.29.0). To assess associations between morphometry, metabolic measures, pasture scores, and the development of ID, a repeated measures mixed effects model was used (SPSS v.29.0), with the pony as the subject variable and time (week) as the repeated measure. The same approach was used to investigate the association between the expression of AdipoR1/2 and [adiponectin]. In all analyses, *p* ≤ 0.05 was considered to indicate statistical significance.

## 3 RESULTS

### 3.1 Morphometry

#### 3.1.1 Body condition and cresty neck scores

**FIGURE 1:**
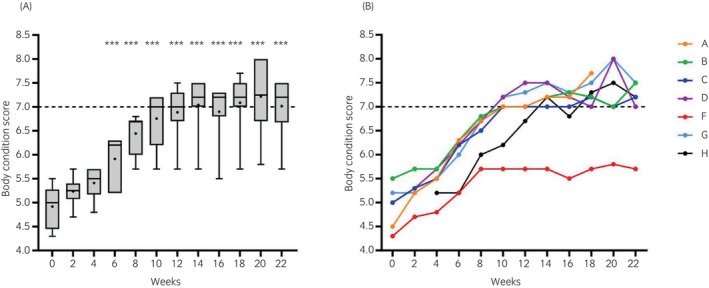
Overall body condition scores (scored out of 9 according to the Kohnke‐modified Henneke scale)^14^, ^17^ from week 0 to 22. *n* = 6 for weeks 0, 2, 20, and 22; *n* = 7 for all other weeks. (A) Median and range (box and whisker) with means shown as dots. (B) individual ponies denoted A–H. ***p ≤ 0.001 relative to week 0.

BCS and CNS increased in all ponies over weeks 0–12, and *n* = 6 ponies reached BCS = 7/9 during the course of the study. BCS was significantly (*p* < 0.001) higher at weeks 6–22 than at week 0 (Figure 1).

Median (range) CNS increased from 2 (1–2; week 0) to 2.5 (2–3; week 12) and 3 (2–3; week 22) and was significantly higher at weeks 4–22 than week 0 (*p* < 0.05; Figure S1).

#### 3.1.2 Other morphometric measures

Scores assigned to each body area are shown in Figure S2 and fortnightly and cumulative changes in bodyweight, heart girth, belly girth, and rump width are shown in Figure S3. Changes in bodyweight were positively correlated with changes in belly girth (Spearman's *ρ* = 0.900) and heart girth (Spearman's *ρ* = 0.709; *p* < 0.05 for both). Fortnightly changes in heart girth, belly girth, rump width, and weight were not significantly correlated with changes in BCS. The percentage changes in weight, belly girth, heart girth, and rump width from week 0 to the time at which BCS = 7/9 were also investigated in the six ponies that reached BCS = 7/9. Percentage changes in rump width showed the most variability between animals (CV = 76.9%). Changes in belly girth were larger and showed lower variability (CV = 25.0%) than changes in heart girth (CV = 52.8%). Belly girth decreased during the obesity maintenance period (weeks 12–22).

### 3.2 Metabolic parameters

#### 3.2.1 Basal insulin and insulin response to oral carbohydrates (OST)

**FIGURE 2:**
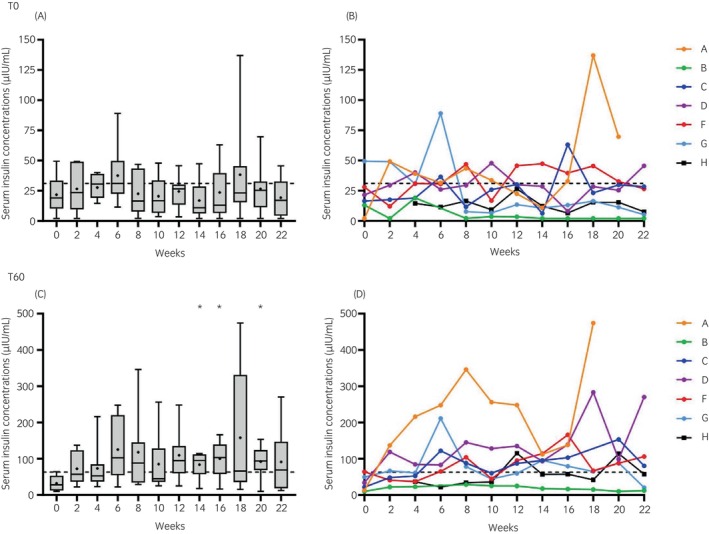
Serum insulin concentrations at T0 (A, B) and T60 (C, D) after oral administration of 0.45 mL/kg Karo Light Corn syrup (oral sugar test). *n* = 6 for weeks 0, 2, 20, and 22; *n* = 7 for all other weeks. *p < 0.05 compared with week 0. (A) Median and range (box and whisker) with means shown as dots. (B) Individual ponies denoted A–H. Dotted lines represent threshold values of T0 = 31 μIU/mL and T60 = 63 μIU/mL for ID (Immulite 2000 xpi).^15^

T0 [insulin] did not change significantly throughout the study (Figure 2A,B). There were 79 basal insulin measurements recorded and basal hyperinsulinaemia was identified on 20 occasions (25.3%). T60 [insulin] was significantly (*p* < 0.05) higher at weeks 14, 16, and 20 than at week 0 (Figure 2C,D). There were 79 measurements available for T60 [insulin] and post‐OST hyperinsulinaemia was identified on 45 occasions (57.7%).

#### 3.2.2 Tissue insulin sensitivity

**FIGURE 3:**
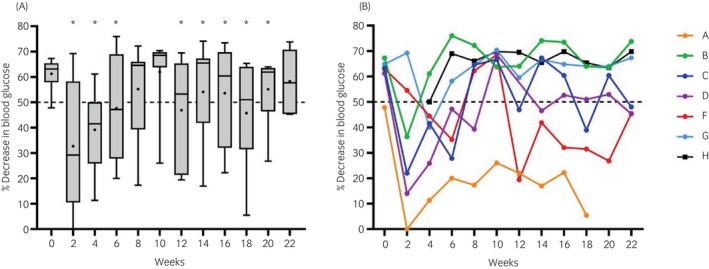
Percentage decrease in blood glucose concentrations recorded 30 min after intravenous administration of 0.1 μIU/kg insulin (insulin tolerance test). *n* = 6 for weeks 0, 2, 20, and 22; *n* = 7 for all other weeks. *p < 0.05 compared with week 0. (A) Median and range (box and whisker) with means shown as dots. (B) Individual ponies denoted A–H.

ITT results were significantly (*p* < 0.05) lower at weeks 2–6 and 12–20 compared with week 0 (Figure 3A). Significant individual variation in ITT results was noted between ponies (Figure 3B). Tissue insulin resistance was identified in 30 of 79 measurements (38.0%).

#### 3.2.3 Adiponectin concentrations

**FIGURE 4:**
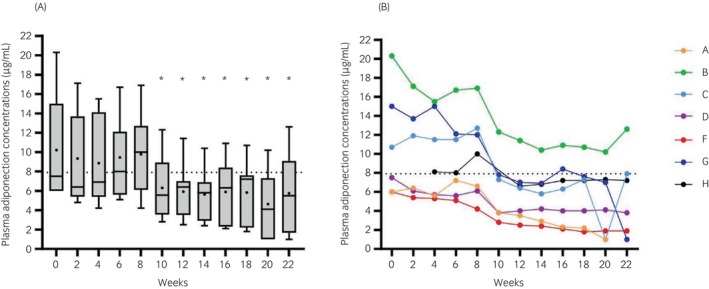
Plasma total adiponectin concentrations measured every 2 weeks from week 0 to week 22. *n* = 6 for weeks 0, 2, 20, and 22; *n* = 7 for all other weeks. *p < 0.05 compared with week 0. (A) Median and range (box and whisker) with means shown as dots. (B) Individual ponies denoted A–H. The dotted line represents [adiponectin] = 7.9 μg/mL, which is the cut‐off value for hypoadiponectinaemia.^15^

Plasma [adiponectin] was significantly (*p* < 0.05) lower at weeks 10–22 than at week 0 (Figure 4). A threshold effect was noted at week 10, which corresponds to the time most ponies reached BCS = 7. All but one of the ponies (pony B) showed hypoadiponectinaemia during the study (Figure 4B) and hypoadiponectinaemia was identified in 52 of 79 measurements (65.8%).

In samples collected in weeks 0 to 8, [adiponectin] was measured before (T0) and 60 min after (T60) administration of Karo Light Corn syrup to determine the short‐term effect of oral sugars on circulating adiponectin concentrations. There was no difference in [adiponectin] between matched T0 and T60 samples (Figure S4).

#### 3.2.4 Gene expression

**FIGURE 5:**
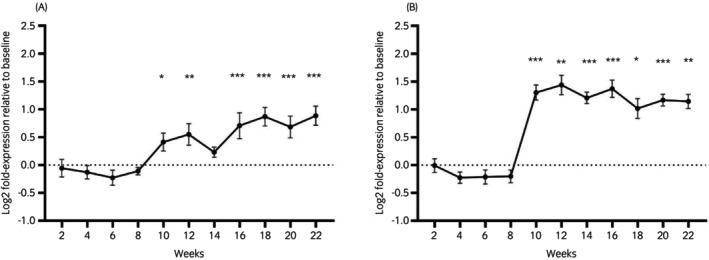
RNA expression of AdipoR1 (A) and AdipoR2 (B) in equine whole‐blood measured over 22 weeks. *n* = 6 for weeks 0, 2, and 22; *n* = 7 for all other weeks. *p < 0.05, **p < 0.01, ***p < 0.001. Data are shown as means ± SD.

RNA expression of AdipoR1 in whole‐blood increased gradually over the study period and was significantly higher at weeks 10, 12, and 16–22 compared with that at week 0 (Figure 5A). Expression of AdipoR2 was significantly higher during weeks 10–22 than at week 0 (Figure 5B), suggesting a threshold effect similar to that observed in [adiponectin]. In a repeated measures model, plasma [adiponectin] was significantly negatively associated with the expression of AdipoR2, but not AdipoR1. There were no significant changes in the expression of INSR or IGF‐1R during the study period (Figure S5).

### 3.3 Pasture scores

**FIGURE 6:**
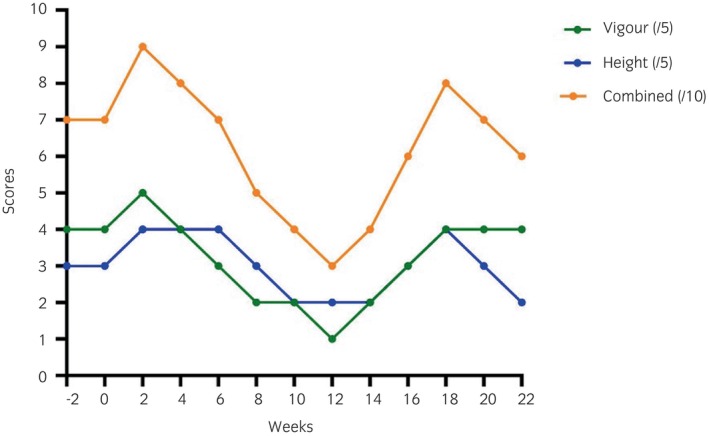
Vigour, height, and combined pasture scores recorded every 2 weeks during the study period (16 May–19 October 2022).

Individual scores for pasture vigour and height (each out of 5) and combined scores (out of 10) are shown in Figure 6. Scores for vigour and height were positively correlated (Spearman's *ρ* = 0.580, *p* = 0.04).

Linear regression was used to investigate the effect of weather conditions (weekly rainfall, as well as monthly sunshine hours, mean maximal temperatures, and mean temperatures) on scores for pasture vigour and height, and combined pasture scores. None of the weather variables investigated here contributed significantly to predicting pasture height scores. However, mean temperature contributed significantly to predicting vigour scores (*F*(1, 11) = 28.55, *p* = 0.001) and combined pasture scores (*F*(1, 11) = 13.79, *p* = 0.003), accounting for 85.0% (adjusted *R* ^2^ = 0.70) and 55.6% (adjusted *R* ^2^ = 0.52) of the variability in vigour and combined pasture scores, respectively. For every 1°C increase in mean temperature, vigour scores and combined pasture scores decreased by 0.40 (95% confidence interval [CI] = 0.57–0.24) and 0.54 (95% CI = 0.86–0.22), respectively.

### 3.4 Development of insulin dysregulation

The ponies' ID status at each time‐point is shown in Table 2. All ponies were classified as being insulin‐dysregulated (ID+) at least once during the study period (mean: 7, range 1–11).

**TABLE 2:** Table showing the ID status of each pony at each time point.

Pony	Weeks												%time pony was ID+
	0	2	4	6	8	10	12	14	16	18	20	22	
A	3	1, 2, 3	1, 2, 3	1, 2, 3	1, 2, 3	1, 2, 3	2, 3	2, 3	1, 2, 3	1, 2, 3			100.0
B		3											9.1
C		3	3	1, 2, 3	2		2, 3	2	1, 2	3	2	2, 3	83.3
D		2, 3	1, 2, 3	2, 3	2	1, 2	2	2, 3	2	2	2	1, 2, 3	91.7
F	2		3	2, 3	1, 2, 3		1, 2, 3	1, 2, 3	1, 2, 3	1, 2, 3	1, 2, 3	2, 3	83.3
G		1, 2	3	1, 2	2, 3			2	2	2	2		66.7
H							2				2		20.0

*Note*: ID+: insulin‐dysregulated, defined as the pony having one of three manifestations of ID represented by numbers: (1) T0 [insulin] >31 μIU/mL OR (2) T60 [insulin] >63 μIU/mL (oral sugar test) OR (3) decrease in blood glucose <50% (insulin tolerance test). Green blocks, not insulin‐dysregulated; orange, insulin‐dysregulated; white, data not available.

#### 3.4.1 Morphometry

Associations between morphometric measures and T0 [insulin], T60 [insulin], ITT results, and [adiponectin] determined using repeated measures models are shown in Table 3. Weight was associated with all analytes and BCS was associated with all except [adiponectin]. Interestingly, rump width was significantly associated only with [adiponectin], suggesting this metric may be useful to track in ponies at pasture. Belly girth was not significantly associated with any of the blood analytes.

**TABLE 3:** Association between various morphometric measures and T0 [insulin], T60 [insulin], insulin tolerance test (ITT) results, and plasma [adiponectin] in repeated measures models.

	T0 [insulin]	T60 [insulin]	ITT results	[Adiponectin]
BCS	*F* (1,22) = 4.549	*F* (1,22) = 4.236	*F* (1,22) = 2.685	NS
	*p < 0.001*	*p < 0.001*	*p = 0.002*	
CNS	NS	*F* (1,4) = 3.263	NS	NS
		*p = 0.023*		
Heart girth	NS	*F* (1,39) = 2.174	*F* (1,40) = 2.158	NS
		*p = 0.009*	*p = 0.047*	
Belly girth	NS	NS	NS	NS
Rump width	NS	NS	NS	*F* (1,24) = 1.931
				*p = 0.038*
Weight	*F* (1,60) = 6,422,092.18	*F* (1,59) = 31.27	*F* (1,61) = 2051.05	*F* (1,60) = 6.352
	*p < 0.001*	*p < 0.001*	*p < 0.001*	*p = 0.013*

*Note*: Results from independent repeated models with pony number as the subject variable and time (weeks) as the repeated measure (SPSS v.29.0).

Abbreviations: BCS, body condition score; CNS, cresty neck score; NS, not significant; ITT, insulin tolerance test.

#### 3.4.2 Adiponectin

[Adiponectin] was a significant variable in a repeated measures model for ITT results only (*F* [1, 57] = 199.845, *p* < 0.001), where higher [adiponectin] was associated with higher blood glucose clearance in the ITT.

#### 3.4.3 Pasture scores

Combined pasture scores were a significant variable in repeated measures models of [adiponectin] (*F* [1, 6] = 3.282; *p* = 0.008) and ITT results (*F* [1, 6] = 5.783; *p* < 0.001), but not T0 or T60 [insulin]. Very low (3/10) and high (8–9/10) combined pasture scores were associated with tissue IR (low ITT results), while low pasture scores (3–4/10) were associated with low [adiponectin].

## 4 DISCUSSION

In this study, seven healthy, non‐laminitic, non‐hyperinsulinaemic ponies at pasture were monitored over late spring/summer, with a focus on morphometry, metabolic indicators, and grazing conditions. All ponies in this cohort developed ID at some point during the study, and ID status varied throughout, sometimes changing from week to week, although these changes were not consistent across ponies. In addition, six ponies showed hypoadiponectinaemia during the study.

Tissue IR and post‐OST hyperinsulinaemia were the most common manifestations of ID and each occurred without the other two manifestations, the third being basal hyperinsulinaemia, at certain time points. Evidence of ID was consistent in five of the ponies but appeared to be transient in the remaining two animals (ponies B and H). Notably, ITT results showed a marked decrease from week 0 to 2 in all ponies. Thereafter, tissue IR recovered in all animals from week 2 to 6 and showed greater individual variability thereafter. This could indicate an adaptive response to counterbalance the tissue IR observed at week 2. Hyperinsulinaemia and tissue IR result from different pathophysiological processes and their relationship remains incompletely understood.^2^ It has been reported previously that ponies may show only one of these two main forms of ID.^22^ The present study supports these findings and goes further in showing that these different pathologies may present at different times within the same animal over a short period of time, with some ponies alternating between IR and hyperinsulinaemia in consecutive tests.

It is also worth noting that there is an incomplete overlap between different tests used to diagnose ID (basal [insulin], post‐OST [insulin], and response to ITT), as was recently investigated by Clark et al. using a Bayesian approach.^22^ Measurement of basal [insulin] had the lowest sensitivity but highest specificity for the diagnosis of ID, while post‐OST [insulin] showed greater sensitivity but lower specificity. The ITT showed both good sensitivity and specificity. In the present study, basal hyperinsulinaemia was never observed without at least one other manifestation of ID. Like those of Clark et al.,^22^ our results support the use of dynamic insulin testing as a more accurate indicator of insulin sensitivity for the diagnosis of ID in clinical practice. Although the reproducibility of the OST has been investigated,^18^, ^27^ there is no data on the repeatability of the ITT in the same animal.

During the study period, [adiponectin] decreased in all ponies. This decrease was gradual at first, and a marked drop was observed from week 8 to 10. Decreases in [adiponectin] became statistically significant at week 10 (last week of July), which is the time at which most ponies reached BCS ≥7. These results support those previously published by Staub et al.,^28^ who reported decreasing [adiponectin] in ponies at pasture from April to October. There has been no study investigating seasonal changes in circulating [adiponectin] with regular repeated measures in equids. In humans, adiponectin shows diurnal,^29^ but not seasonal variation.^30^, ^31^ Longitudinal studies tracking changes in [adiponectin] over time would be very useful in determining seasonal variation as well as association with long‐term health outcomes in ponies.^32^

The physiological processes linking ID and hypoadiponectinaemia are complex and incompletely understood. Adiponectin is a key insulin‐sensitising agent that activates the catabolic, ATP‐producing adenosine monophosphate kinase (AMPK) pathway, which is an important balance to the anabolic mammalian target of rapamycin (mTOR) pathway promoting cell proliferation.^33^ Evidence of the involvement of both these pathways in the pathophysiology of laminitis was recently reviewed.^34^ In conditions of hypoadiponectinaemia and consequent reduced AMPK pathway activation, it is hypothesised that the energy balance is shifted toward increased mTOR activation, which is further exacerbated by hyperinsulinaemia and inappropriate activation of the IGF‐1 receptors by insulin.^13^ This hypothesis, however, remains to be investigated in equine in vitro and in vivo models. Although hyperinsulinaemia and hypoadiponectinaemia are both independent risk factors for endocrinopathic laminitis, it is unclear whether one is a cause or consequence of the other, and a previous study reported that short‐term experimentally induced hyperinsulinaemia did not lead to hypoadiponectinaemia.^11^

This is further complicated by the inconsistent relationship between obesity and ID, which also affect and are affected by adiponectin signalling. The inverse correlation between adiponectin secretion and obesity is thought to be a result of multiple cellular processes,^32^ including changes in adipocyte function associated with hypertrophy, chronic inflammation, and hypoxia.^35^ Reports of correlations between BCS, CNS, and adiponectin concentrations are inconsistent^6^, ^7^, ^9^ and neither BCS nor CNS were significant factors in a repeated measures model of [adiponectin] in this study. It is worth noting that the one pony who failed to become obese also showed a decrease in [adiponectin] similar to that in the other, obese ponies.

Although elucidating the pathophysiology of ID and hypoadiponectinaemia was not possible based on the results of the present study, we can conclude that any pony may show decreased adiponectin concentrations when turned out to pasture, regardless of ID status, obesity, or clinical laminitis. In addition, there appears to be an important inherent factor or individual ‘starting point’ for adiponectin concentrations. Ponies with high or low baseline [adiponectin] tended to maintain high or low concentrations throughout the study period, respectively, with some variability that may be associated with obesity and ID. This raises questions as to whether and to what extent adiponectin concentrations may be modifiable in equids.

Expression of the two main adiponectin receptors was investigated in whole‐blood to build on previous research investigating these receptors in adipose tissue^28^ and to provide a more complete understanding of signalling via circulating adiponectin. Furthermore, because of the signalling pathways activated by each of these receptors and their affinity for different forms of adiponectin, some insight may be gained into the action and regulation of adiponectin isoforms for which there are no validated assays available in the UK currently. There were significant increases in the RNA expression of both AdipoR1 and AdipoR2 in whole‐blood during the study period, with greater upregulation of AdipoR2. AdipoR1 shows greater affinity for the globular isoform of adiponectin while AdipoR2 binds primarily full‐length adiponectin.^36^ Considering that increased expression of receptors may be a compensatory response to the decrease in circulating [adiponectin] (i.e., physiologically expected receptor up‐regulation), these results possibly indicate a greater decrease in full‐length adiponectin. Direct measurement of different adiponectin isoforms would be required to investigate this hypothesis. Furthermore, AdipoR2 activation is associated with peroxisome proliferator‐activated receptor (PPAR)‐α signalling, while AdipoR1 primarily activates the AMPK pathway.^37^, ^38^ Differences in the upregulation of both receptors therefore may indicate differential regulation of these two pathways. Further research should be done to investigate this in in vitro models of equine cells and tissues. Staub et al.^28^ showed a decrease in adiponectin expression and a corresponding increase in the expression of AdipoR1 and AdipoR2 in the adipose tissue of Welsh ponies from April to July, concurrent with pasture‐induced weight gain. However, RNA rather than protein expression was measured in the present study and in that by Staub et al.,^28^ so it remains unclear whether these changes in expression are reflected in active tissue receptors.

Potential associations between grazing, morphometric measures, metabolic indicators, and manifestations of ID were investigated, but no clear factors predictive of the development of ID were identified. Heart girth measurements were significantly associated with post‐OST hyperinsulinaemia and tissue IR. In addition, changes in belly girth and heart girth were strongly positively correlated with weight gain, suggesting that these two measurements may be useful for regular monitoring of weight gain/loss when a weighbridge is not available, as previously suggested.^39^ Belly girth showed large changes during the weight gain phase and decreased during the obesity maintenance period, potentially suggesting changes in body fat composition or gut fill. Accordingly, belly girth may be a more reliable indicator of initial weight gain (and weight loss)^40^ rather than a metric for monitoring obesity over the long term. However, it is important to note that although these measures may be useful in tracking weight gain, they appear to be unreliable indicators of changes in basal [insulin] and [adiponectin].

Using the pasture scoring system devised in this study, combined pasture scores were a significant factor in repeated measures models of ITT results and [adiponectin]. Specifically, results suggest that both short, stressed grass (score 3/10) and very long lush grass with high growth (score 8–9/10) were associated with tissue IR. Stressed grass is likely to have a high non‐structural carbohydrate (NSC) content per blade of grass, whereas long, lush grass is likely to provide greater NSC content per mouthful or per day because of its greater herbage yield, both of which could result in higher NSC intake.^41^, ^42^ The simple pasture assessment tool developed in the present study shows promise and may be useful for horse‐owners and other laypersons but will need further validation in larger cohort studies.

Although all ponies were exposed to the same environmental risk (pasture) in this study, all had different responses in terms of weight gain and changes in ID status. Some animals became ID+ very soon after being turned out to pasture and remained ID+ throughout, while others remained non‐ID for most of the study despite showing substantial weight gain. One pony failed to become obese during the study but was ID+ and consistently hypoadiponectinaemic throughout the study period. This inherent variation between ponies likely points to the importance of a genetic predisposition to ID,^43^, ^44^, ^45^ although this was not explored in the current study. Other factors likely to be relevant but not considered in this study include individual characteristics such as the animal's gut microbiome^46^, ^47^, ^48^ and enteroinsular axis,^49^ as well as the composition and nutritional value of the pasture. Although all ponies in this study were grazing in the same location, actual NSC intake is likely to vary between different animals according to behaviour including foraging efficiency,^50^ social dominance,^51^ time of day at which the pony is grazing,^42^, ^52^ and even choice of grazing area.^42^, ^53^

Two of the eight ponies developed mild laminitis during the study, despite both having normal basal and post‐OST [insulin] concentrations at the time of recruitment to the study and no clinical signs of previous laminitis. One of these ponies had BCS = 5.0 at the onset of laminitis, which would be considered ‘ideal’ bodyweight. Therefore, although non‐laminitic animals would not typically undergo regular [insulin] testing when turned out to pasture, especially if they do not show weight gain, this study suggests that this is a valuable approach for any animals with predisposing risk factors (e.g., breed or activity level).^54^ In the present study, animals were monitored daily, which enabled signs to be picked up very quickly, and this confirms the necessity to carefully evaluate even ponies deemed at low risk when at pasture.

This study has several limitations. The first and most significant of these is the lack of a control group in which animals would have been kept at the same pasture but maintained at ideal bodyweight to determine normal seasonal variations in ID status and plasma [adiponectin] not associated with weight gain. Without this, it is impossible to distinguish the effects of pasture consumption and obesity. Although this would have been the best approach scientifically, keeping ponies at pasture for 22 weeks over spring/summer without inducing any weight gain would have presented very significant practical challenges. Second, this was a small sample size, all of which were native UK pony breeds. Although the study aimed to include eight ponies, one was removed early on and was not included in subsequent analysis. There was marked variation in the data at certain time‐points, and this high background noise could have masked some of the findings, which may be revealed more clearly with a larger sample size. Third, although ponies were considered non‐hyperinsulinaemic at the time of recruitment to the study, [adiponectin] and tissue IR were not assessed as part of pre‐recruitment screens. One of the ponies (A) showed IR at week 0 and all subsequent weeks. This animal may already have been IR at the time of recruitment, and performing an ITT at this time would have confirmed this. Finally, pasture analyses were outside the scope of this study but could have provided valuable data. This includes measurement of the pasture's NSC content and its variability over time, as high NSC content exacerbates hyperinsulinaemia and insulin resistance.^55^, ^56^ This would also have permitted correlation analysis of the qualitative pasture scoring system developed here and the quantitative NSC content. Another important consideration that was outside the scope of this study is data relating to the ponies' physical activity. This could have been collected using wearable accelerometers^57^ and may have provided an indication of why pony H did not gain weight.

In conclusion, this study revealed that ponies undergoing weight gain at pasture show high variability in ID status over time and between individual animals. Despite having normal basal and post‐OST [insulin] at the start, all developed transient or consistent ID during the study. Tissue IR and post‐OST hyperinsulinaemia were the most common manifestations of ID, and each occurred without the other two manifestations at certain time points. All ponies showed decreased [adiponectin] when compared with week 0, although it is unclear whether this was a normal seasonal change or was associated with weight gain. Changes in grazing conditions were associated with tissue IR and [adiponectin] with both short, stressed grass and very long lush grass being associated with lower ITT results. The use of the simple pasture scoring system developed here warrants further investigation for use by horse‐owners and carers.

## FUNDING INFORMATION

This study was supported by grants from Waltham Petcare Science Institute and the Royal Veterinary College Mellon Fund.

## CONFLICT OF INTEREST STATEMENT

Pat Harris is an employee of Waltham Petcare Science Institute.

## AUTHOR CONTRIBUTIONS

**Marine A. Barnabé:** Conceptualization; formal analysis; writing – review and editing; writing – original draft; investigation. **Jonathan Elliott:** Conceptualization; funding acquisition; writing – review and editing; supervision. **Patricia A. Harris:** Conceptualization; funding acquisition; writing – review and editing; supervision. **Nicola J. Menzies‐Gow:** Conceptualization; investigation; funding acquisition; writing – review and editing; supervision.

## DATA INTEGRITY STATEMENT

M. Barnabé had full access to all the data in the study and takes responsibility for the integrity of the data and the accuracy of data analysis.

## ETHICAL ANIMAL RESEARCH

This study was approved by the Royal Veterinary College Animal Welfare and Ethical Review Board (2020‐135N) and the Clinical Research Ethical Review Board (URN 2022‐2138‐2) and was conducted under a UK Home Office licence (PP5634400).

## INFORMED CONSENT

Not applicable.

## ANTIMICROBIAL STEWARDSHIP POLICY

Not applicable.

## Supporting information


**Figure S1.** Cresty neck scores recorded from weeks 0 to 22.

**Figure S2.** Body condition scores assigned to each body area from weeks 0 to 22.

**Figure S3.** Fortnightly and cumulative changes (%) in bodyweight, rump width, belly girth, and heart girth.

**Figure S4.** Plasma total adiponectin concentrations measured before (T0) and 60 min after (T60) administration of 0.45 mL/kg Karo Light Corn syrup.

**Figure S5.** RNA expression of insulin‐like growth factor 1 receptor (A) and insulin receptor (B) in equine whole‐blood measured over 22 weeks.

**Data S1.** Supporting Information.

**Table S1.** Characteristics of animals recruited to the study.

## Data Availability

The data that support the findings of this study are openly available in Zenodo (https://doi.org/10.5281/zenodo.14536573), reference number 14536573.
