# Supplementary material for: Effects of pasture consumption and obesity on insulin dysregulation and adiponectin concentrations in UK native‐breed ponies
Source: Equine Vet J. 2025 Apr 21;58(1):243–55. doi: 10.1111/evj.14507 (PMC12699113; doi:10.1111/evj.14507)
Supplement: Supplementary file 1 — Figure S1. Cresty neck scores recorded from weeks 0 to 22. [file EVJ-58-243-s007.pdf]

Equine Veterinary Journal

BEVA

Figure S1: Cresty neck scores recorded from weeks 0 to 22.

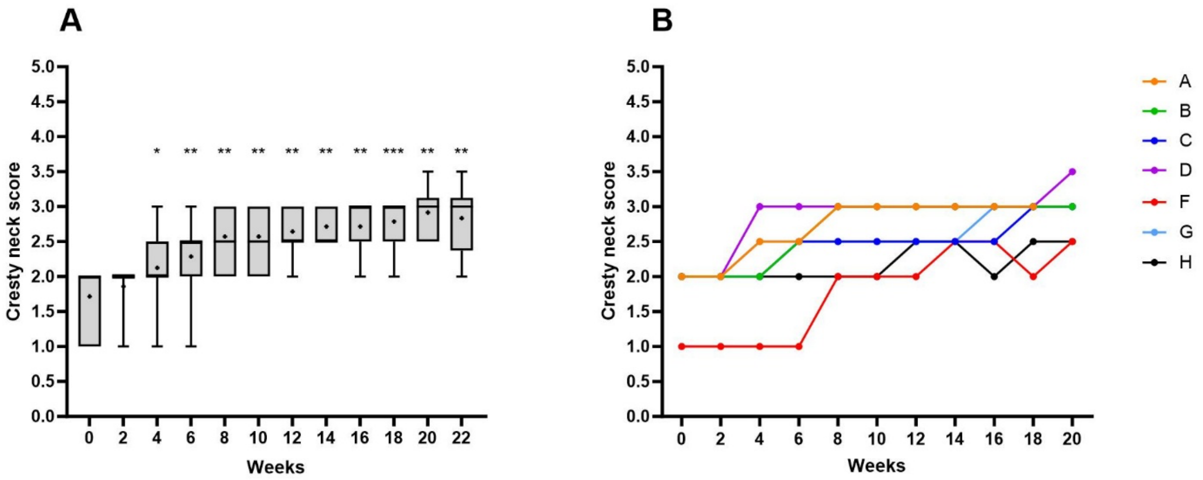

n = 6 for weeks 0, 2, 20, and 22; n = 7 for all other weeks.
A, median and range (box and whiskers) with means shown as dots.
B, individual ponies denoted A–H. *P ≤ 0.05; **P ≤ 0.01; ***P ≤ 0.001 relative to week 0.
