# Supplementary material for: Effects of pasture consumption and obesity on insulin dysregulation and adiponectin concentrations in UK native‐breed ponies
Source: Equine Vet J. 2025 Apr 21;58(1):243–55. doi: 10.1111/evj.14507 (PMC12699113; doi:10.1111/evj.14507)
Supplement: Supplementary file 2 — Figure S2. Body condition scores assigned to each body area from weeks 0 to 22. [file EVJ-58-243-s004.pdf]

Figure S2: Body condition scores assigned to each body area from weeks 0 to 22.

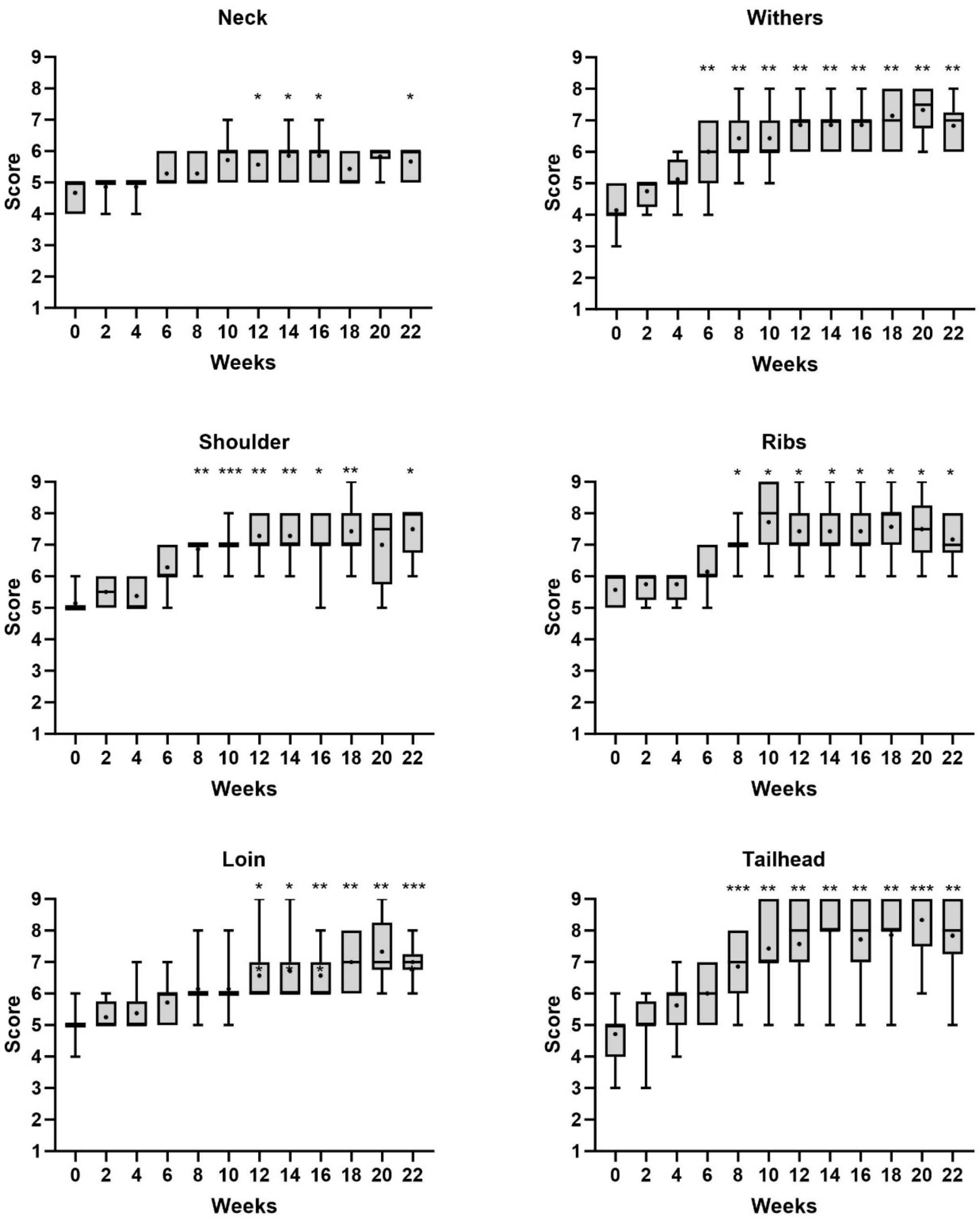

n = 6 for weeks 0, 2, 20, and 22; n = 7 for all other weeks.
Data are presented as median and range with means showed as dots. *P ≤ 0.05; **P ≤ 0.01; ***P ≤ 0.001 relative to week 0.
