# Supplementary material for: Effects of pasture consumption and obesity on insulin dysregulation and adiponectin concentrations in UK native‐breed ponies
Source: Equine Vet J. 2025 Apr 21;58(1):243–55. doi: 10.1111/evj.14507 (PMC12699113; doi:10.1111/evj.14507)
Supplement: Supplementary file 3 — Figure S3. Fortnightly and cumulative changes (%) in bodyweight, rump width, belly girth, and heart girth. [file EVJ-58-243-s003.pdf]

Equine Veterinary Journal BEVA

Figure S3: Fortnightly and cumulative changes (%) in bodyweight, rump width, belly girth, and heart girth.

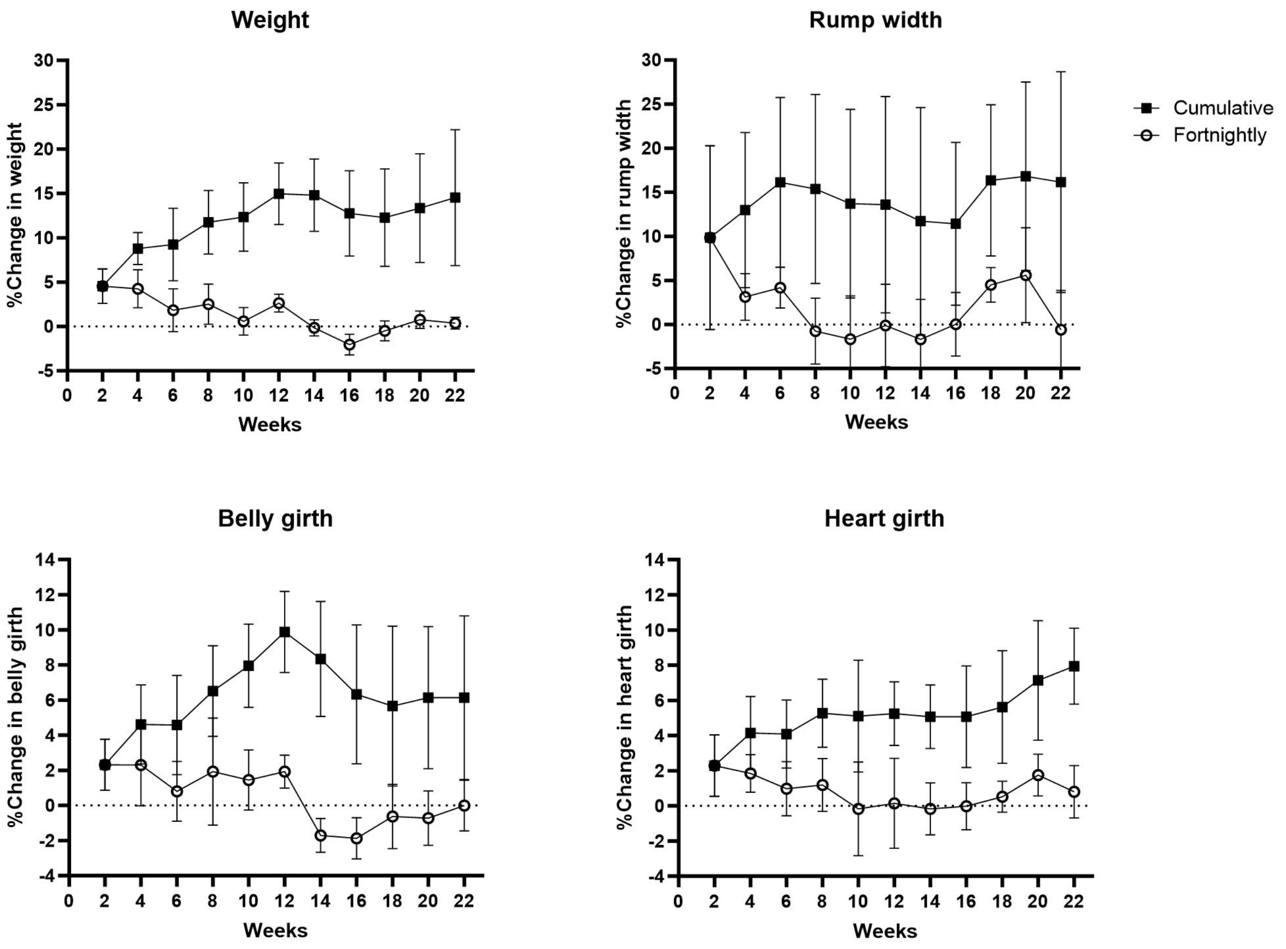

n = 6 for weeks 0, 2, 20, and 22; n = 7 for all other weeks.
Data are presented as means ± SD.
