# Supplementary material for: Effects of pasture consumption and obesity on insulin dysregulation and adiponectin concentrations in UK native‐breed ponies
Source: Equine Vet J. 2025 Apr 21;58(1):243–55. doi: 10.1111/evj.14507 (PMC12699113; doi:10.1111/evj.14507)
Supplement: Supplementary file 4 — Figure S4. Plasma total adiponectin concentrations measured before (T0) and 60 min after (T60) administration of 0.45 mL/kg Karo Light Corn syrup. [file EVJ-58-243-s002.pdf]

Equine Veterinary Journal BEVA

Figure S4: Plasma total adiponectin concentrations measured before (T0) and 60 min after (T60) administration of 0.45 mL/kg Karo Light Corn syrup.

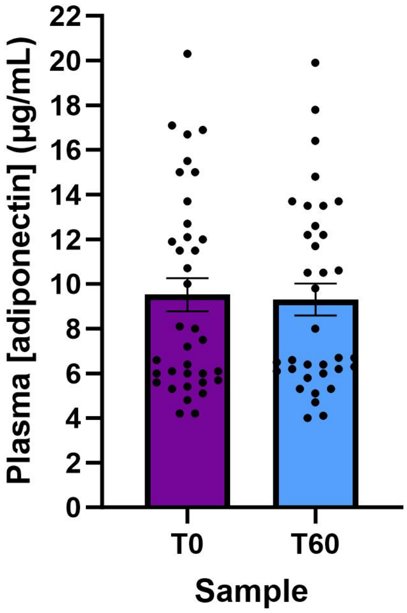

Data are presented as means ± SEM from 35 data-points.
