# Supplementary material for: Effects of pasture consumption and obesity on insulin dysregulation and adiponectin concentrations in UK native‐breed ponies
Source: Equine Vet J. 2025 Apr 21;58(1):243–55. doi: 10.1111/evj.14507 (PMC12699113; doi:10.1111/evj.14507)
Supplement: Supplementary file 5 — Figure S5. RNA expression of insulin‐like growth factor 1 receptor (A) and insulin receptor (B) in equine whole‐blood measured over 22 weeks. [file EVJ-58-243-s001.pdf]

Equine Veterinary Journal BEVA

Figure S5: RNA expression of insulin-like growth factor 1 receptor (A) and insulin receptor (B) in equine whole-blood measured over 22 weeks.

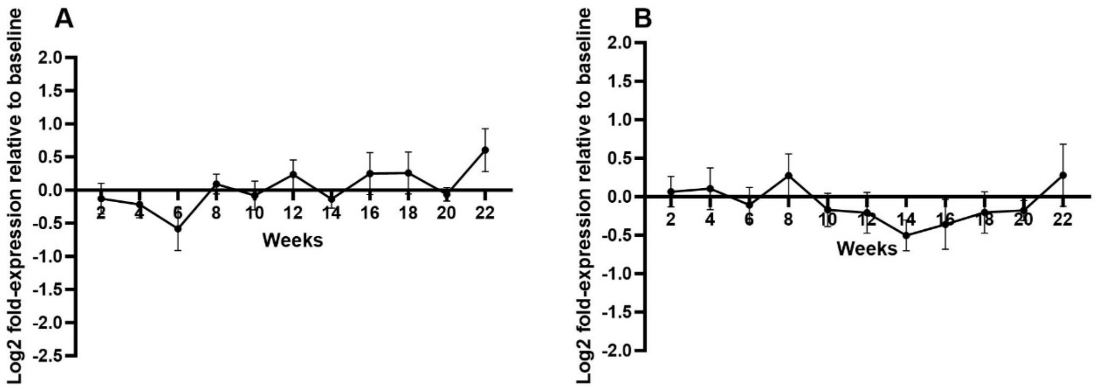

n = 6 for weeks 0, 2, and 22; n = 7 for all other weeks. *P < 0.05, **P < 0.01, ***P < 0.001.
Data are shown as means ± SD.
