# Supplementary material for: Effects of pasture consumption and obesity on insulin dysregulation and adiponectin concentrations in UK native‐breed ponies
Source: Equine Vet J. 2025 Apr 21;58(1):243–55. doi: 10.1111/evj.14507 (PMC12699113; doi:10.1111/evj.14507)
Supplement: Supplementary file 6 — Data S1. Supporting Information. [file EVJ-58-243-s005.docx]

**Efectos del consumo de pasto y obesidad sobre la desregulación insulínica y concentraciones de adiponectina en razas de ponis nativos al Reino Unido.**

Marine A. Barnabé,^1^* Jonathan Elliott,^2^ Pat A. Harris,^3^ Nicola J. Menzies-Gow^1^

^1^Department of Clinical Sciences and Services, Royal Veterinary College, Hawkshead Lane, North Mymms, Hertfordshire, AL9 7TA, RU

^2^Department of Comparative Biomedical Sciences, Royal Veterinary College, Hawkshead Lane, North Mymms, Hertfordshire, AL9 7TA, RU

^3^Equine Studies Group, Waltham Petcare Science Institute, Freeby lane, Waltham-on-the-Leics, LE 14TRT, RU

*Autor corresponsal: [mbarnabe@rvc.ac.uk](mailto:mbarnabe@rvc.ac.uk)

Este estudio fue llevado a cabo en el Royal Veterinary College, Hawkshead Lane, North Mymms, Hertfordshire, AL9 7TA, RU.

**Palabras clave**: adiponectina, clasificación de condición corporal, desregulación insulínica, síndrome metabólico equino, poni, laminitis

**Resumen**

**Historial**: La desregulación insulínica (ID) y la hipoadiponectinemia ([adiponectina]total < 7.9 µg/mL) son factores de riesgo para laminitis. Se asocian, algunas veces, pero no siempre, con obesidad.

**Objetivos**: Investigar los efectos del consumo de pasto y obesidad sobre la ID y la [adiponectina] total en ponis.

**Diseño del estudio**: Longitudinal.

**Métodos**: Siete ponis de razas nativas con una prueba oral basal y post glucosa normales (OST) de [insulina] y clasificación de condición corporal (BCS) 4.3–5.5/9, fueron permitidos pastar hasta que llegasen a tener un BCS 7/9. Los ponis luego fueron mantenidos en BCS 7/9 hasta completar el estudio (semana 22). Medidas morfométricas, OST, prueba a la tolerancia a la insulina (ITT), [adiponectina] plasmática, expresión de receptores en sangre entera a adiponectina, insulina, and factor 1 de crecimiento tipo insulina, y condiciones del pasto (altura y vigor) fueron medidos cada 2 semanas

**Resultados**: La mediana de los BCS (rango) aumento significativamente (P<0.001) de 5.0 (4.3–5.5; semana 0) a 7.2 (5.7–7.5; semana 22). [Insulina] basal no cambio significativamente durante el estudio pero la mediana post-OST [insulina] fue significativamente mayor (P<0.05) en la semana 14 (95.2 [17.9–114.0] µIU/mL), semana 16 (103.0 [16.4–166.0] µIU/mL), y semana 20 (93.6 [10.0–153.0] µIU/mL) que la semana 0 (25.0 [10.0–64.0] µIU/mL). En comparación con la semana 0, los resultados de ITT fueron significativamente menores en las semana 2–6 y 12–20, y [adiponectina] fue significativamente menor a las semanas 10–22 (P<0.05). La [adiponectina] disminuyó en todos los ponis durante el estudio. Tanto los niveles bajos (3/10) y altos (8-9/10) de pasto, fueron significativamente asociados con resultados de ITT bajos. Niveles bajos de pasto fueron asociados con bajas [adiponectina]. Los BCS fueron asociados significativamente con [insulina] basal, [insulina] post-OST, resultados de ITT, pero no con [adiponectina].

**Limitaciones principales**: No hay grupo control con mantención de BCS ideales; tamaño de muestra pequeño que incluye razas nativas del RU.

**Conclusiones**: Seis ponis desarrollaron hipoadiponectemia y todos mostraron una ID transitoria o consistente durante el estudio. Tanto el pasto corto, estresado y aquel largo y frondoso, se asocio con una reducción en la capacidad tisular de respuesta a la insulina.
