# Supplementary material for: Effects of pasture consumption and obesity on insulin dysregulation and adiponectin concentrations in UK native‐breed ponies
Source: Equine Vet J. 2025 Apr 21;58(1):243–55. doi: 10.1111/evj.14507 (PMC12699113; doi:10.1111/evj.14507)
Supplement: Supplementary file 7 — Table S1. Characteristics of animals recruited to the study. [file EVJ-58-243-s006.pdf]

Equine Veterinary Journal BEVA

Table S1: Characteristics of animals recruited to the study.

Pony ID	Sex	Breed	Age (years)	Height (cm)	Plasma ACTH (pg/mL)*	Basal insulin (μIU/mL)†	Post-OST insulin (μIU/mL)†	Number of time-points with data
A	Mare	WMN	14	128	25.7	2.0	31.3	10
B	Mare	Cob	8	134	ND	2.0	5.3	12
C	Gelding	New Forest	6	142	ND	6.8	48.9	12
D	Mare	WMN	12	120	11.9	2.0	54.7	12
E	Mare	WMN	18	117	28.4	2.0	29.1	3
F	Gelding	WMN	5	109	ND	4.8	30.0	12
G	Gelding	WMN	5	104	ND	3.2	30.2	12
H	Gelding	WMN	9	105	ND	7.6	48.8	10

* ACTH was only determined in animals aged > 10 years.
†Determined at the time of recruitment to the study.
ACTH, adrenocorticotropic hormone; ND, not determined; WMN, registered Welsh Mountain Pony.
